# Caution in Giving Albumen as an Antidote

**Published:** 1845-01

**Authors:** 


*Caution in giving Albumen as an Antidote.*—Practitioners, in employing albumen as an antidote to corrosive sublimate, should be aware that it may be given in too great quantity, as the compound formed is soluble in an excess of albumen, and in the deleterious combination which enters the blood, producing the remote influence of the poison. So long as the vomited matters contain a white opaque material admixed, the antidote should not be withheld; when the *ejecta*, on the contrary, become transparent, the further employment of the remedy is generally useless, and may be injurious.

*Dub. Med. Jour.*

